# Supplementary material for: CCR7 Mediates Cell Invasion and Migration in Extrahepatic Cholangiocarcinoma by Inducing Epithelial–Mesenchymal Transition
Source: Cancers (Basel). 2023 Mar 21;15(6):1878. doi: 10.3390/cancers15061878 (PMC10047000; doi:10.3390/cancers15061878)

Figure S1.

Figure 3. (A)

RT-PCR analysis of CCR7 and β-actin mRNA expression in each cell line.

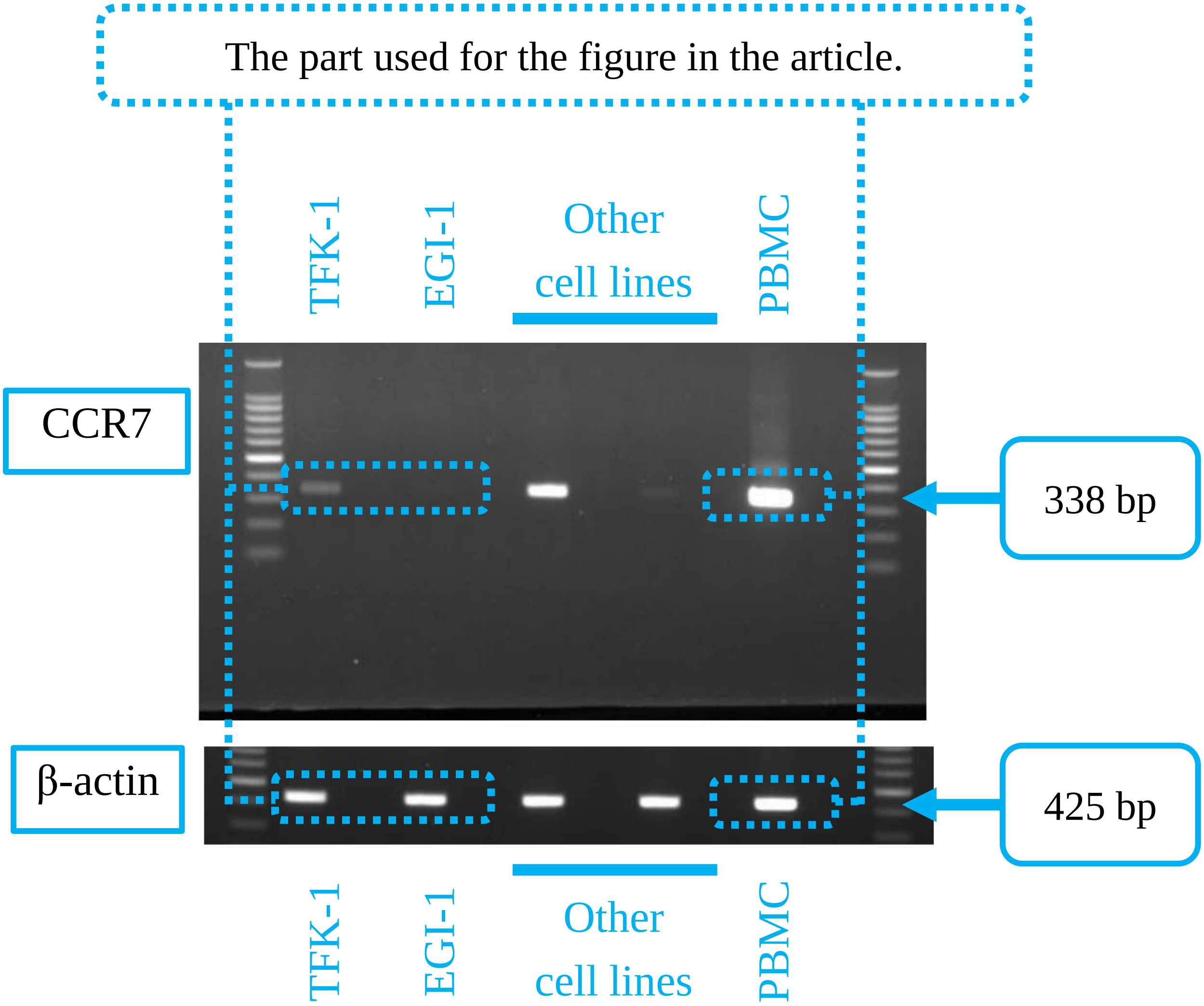

Figure 3. (B) TFK-1

Changes in E-cadherin, vimentin and β-actin protein levels following treatment with CCL19 in TFK-1.

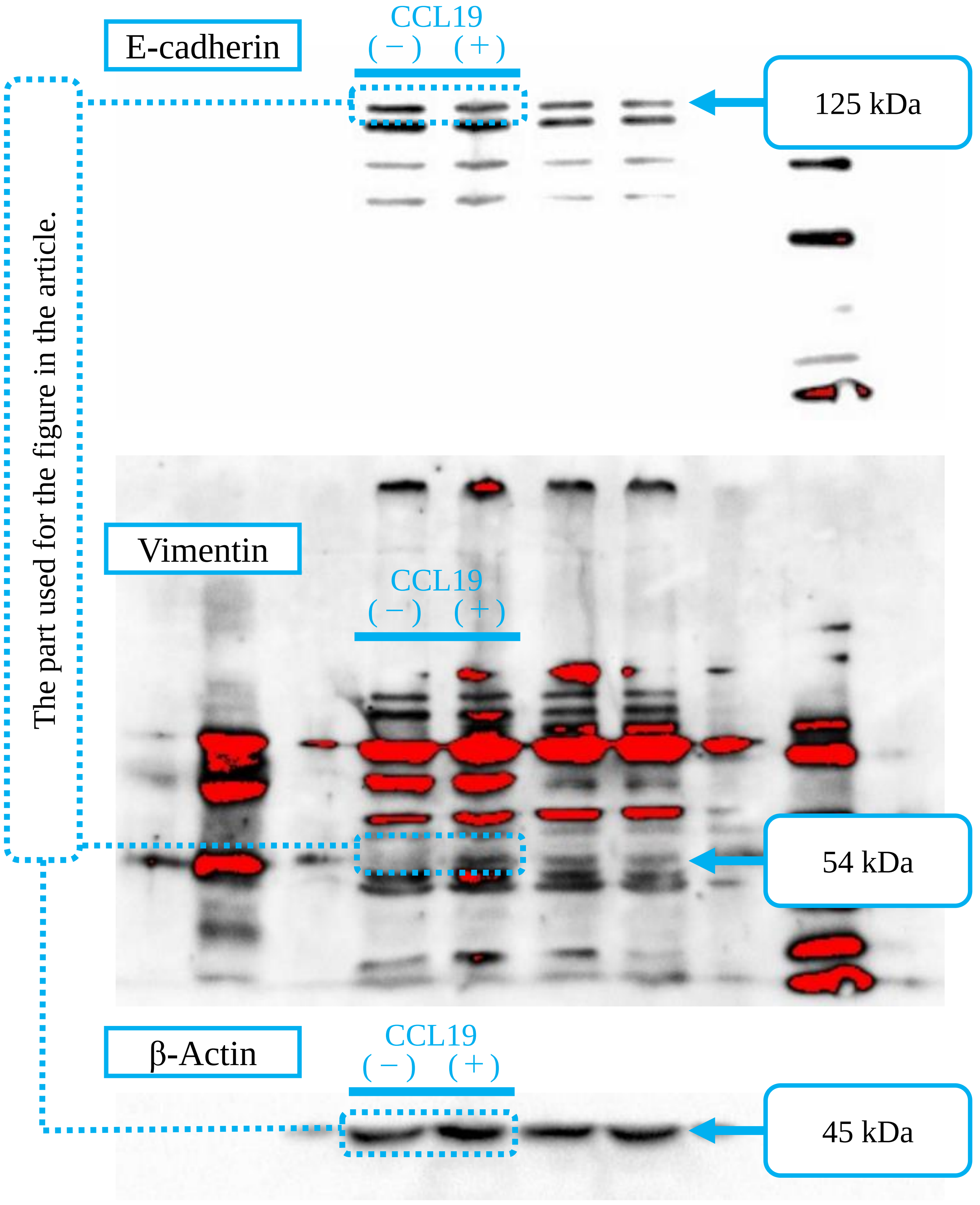

Figure 3. (B) EGI-1

Changes in E-cadherin, vimentin and β-actin protein levels following treatment with CCL19 in EGI-1.

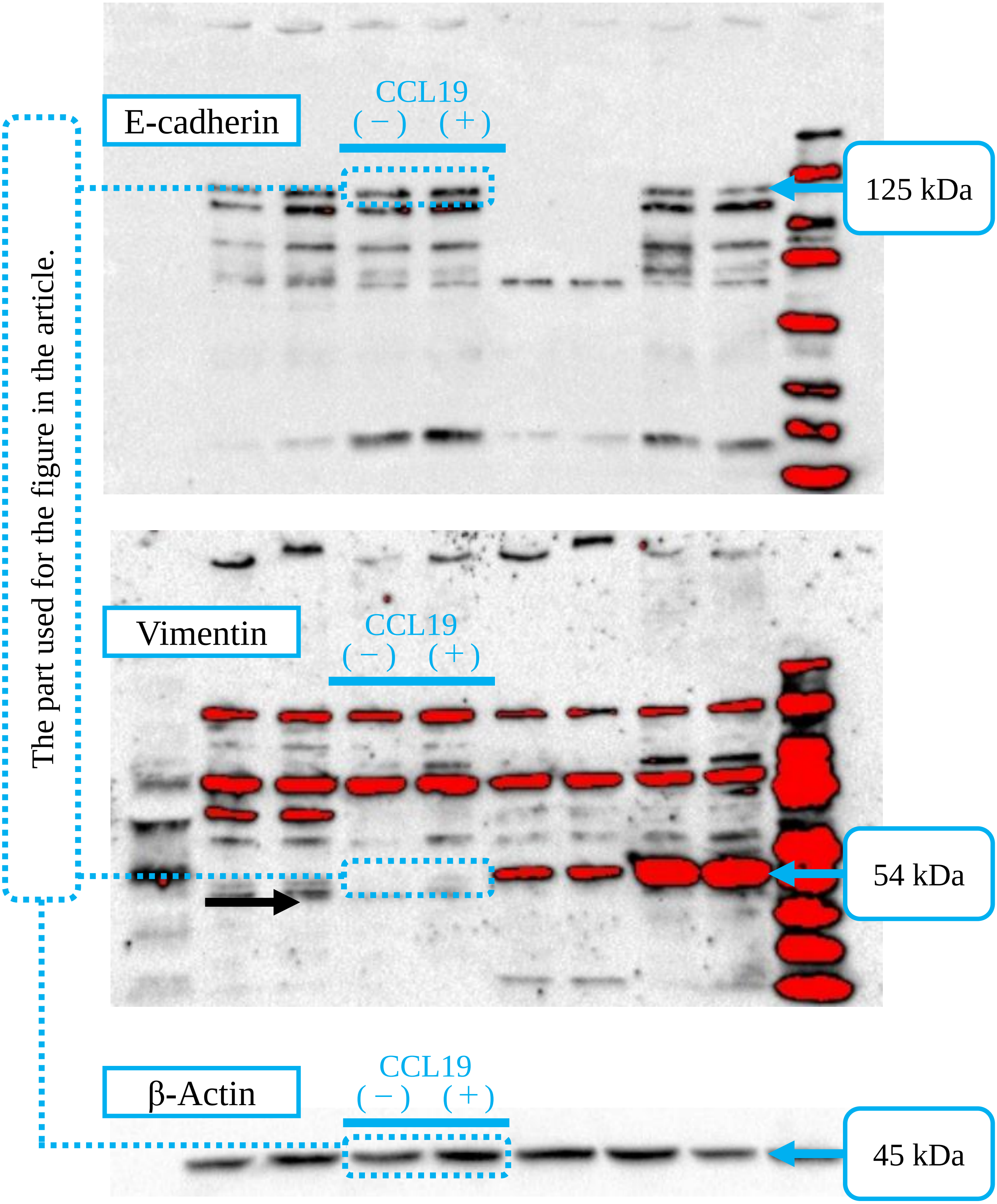

Supplement: Supplementary file 1 [file cancers-15-01878-s001.zip › cancers-2243005-supplementary.pdf]
